# The Investigation of Silicon Localization and Accumulation in Citrus

**DOI:** 10.3390/plants8070200

**Published:** 2019-07-01

**Authors:** Mireille Asanzi Mvondo-She, Diana Marais

**Affiliations:** Department of Plant and Soil Sciences, University of Pretoria, Pretoria 0002, South Africa

**Keywords:** Clementine, energy dispersive X-ray analysis, silicon uptake, Valencia

## Abstract

Several studies have demonstrated Si absorption in monocotyledon and dicotyledon species. Regarding Si accumulation, studies in monocotyledons have identified Si deposition around the cell wall, cuticle layer bulliform cells, silica cells and endodermal cells. In previous studies with different citrus species there were evidence of Si accumulation, however no information on Si deposition can be found. Therefore, in this study, Si was applied (0 and 1000 mg L^−1^) to the roots of two citrus species, ‘Delta’ Valencia (*Citrus sinensis*) and ‘Nules’ Clementine (*Citrus reticulata*). Si accumulation were investigated in new, and old leaves and roots. Si deposition was investigated through scanning electron microscopy using energy dispersive analysis X-ray, environmental scanning electron microscopy and light microscopy. Si accumulation was significantly higher in Si treated leaves comparatively to the control in both young and mature leaves. Meanwhile, Si accumulation increased with leaf age. Additionally, Si accumulation was significantly higher in roots than in leaves. With respect to Si deposition, granules were identified in the epidermal cells through SEM and ESEM studies. The light microscopy identified the presence of Si granules in the surface and around the outer cell surface forming the cuticle-silica double layer of the lower epidermis in Si treated plants. Silica deposit were not found on the abaxial leaf surface. The findings suggest a passive uptake in citrus species.

## 1. Introduction

Silicon (Si) is the second most abundant element in the earth’s crust after oxygen. These two elements are combined in the aluminosilicates of rocks, clays and soil minerals [1]. Plant available Si can be found in the soil solution in an undissociated form as monosilicic acid (Si(OH)_4_) in a concentration range of 90–150 mg L^−1^ in soils with a pH lower than eight [2,3,4]. In highly weathered soils, Si availability in soil solution is reduced considerably because of soil acidification, organic complexes, presence of aluminium, iron and phosphate ions, temperature, sorption/dissolution reaction and soil moisture [4,5]. This leads to the reduction in plant available Si and the need to supplement with silicon fertilizer in order to improve the quality and yield of agricultural crops under abiotic and biotic stress conditions [4,6,7,8].

Silicon is not defined as an essential element for higher plants, although it significantly improves fitness in nature and increases agricultural productivity and is present in plants in amounts equivalent to certain macronutrients such as Ca, Mg and P [9].

There are two general mechanisms for Si uptake and transport (active and passive uptake) co-existing in a plant, with their relative contribution being dependent much upon the plant species and external Si concentration [2,10]. *Oryza sativa* and *Zea mays*, representatives of monocots, have Si in their tissues in the order of 5% or higher (dry weight basis) and are known as Si accumulators. On the other hand, *Helianthus annuus* and *Benincasa hispida*, representatives of dicots, contain about 0.1% Si on a dry weight basis, and are described as intermediate types [3,7]. In another study conducted by [6], Si concentration in the root cell symplast of rice was higher than the external Si in the soil solution. This suggests that silicic acid is taken up against the concentration gradient from the external solution to the cortical cells which results in an active uptake process. Regarding dicots, the coexistence of both a passive and active transport dependent upon the external Si supply was identified in cucumber [10]. While in another study, the passive uptake mechanism were observed in cucumber and tomato based on their root ability and their radial transporters that enables them to take up less Si than rice due to transporter for xylem loading in the latter [6]. This implies that the Si uptake mechanism is also influenced by three transporters identified in higher plants and involved in Si translocation from the xylem and distribution in the leaves [11,12,13,14,15,16,17]. The Si influx transporter Lsi1 is mainly expressed in the main roots and lateral roots but not in root hairs and responsible for the transport of Si from the external solution to the root cells [2,12]. Homologs of Lsi1 have also been reported in barley, maize, pumpkin and wheat [13,18,19]. While, the Si efflux transporter Lsi2 is found in the endodermis of rice roots [12]. Homologs of the Lsi2 transporter have also been reported in barley, maize and pumpkin [13,20]. The transporter Lsi6 is a homolog of the Si influx transporter Lsi1 in rice, barley and maize [11,15]. It is a plasma membrane protein localized at xylem cells of leaf sheaths and blades therefore, it plays a role in the unloading of Si from xylem to leaf tissues [11,20].

Silica deposition in plants is found in outer cell surface which constitutes that the cuticle-silica double layer and has been hypothesized to be dependent on transpiration rate [9,16]. The role of transpiration in Si accumulation implies that Si should be densely deposited in the mesophyll tissue where most of the transpiration takes place [21]. However, Si was deposited in both the mesophyll regions and epidermal cells in Poaceae implying that in addition to the influence of transpiration on the Si uptake, plants also positively controlled the Si accumulation process [22].

Silicon deposition have been found in leaf blades and inflorescence bracts tissues [22,23,24]. Other areas of Si deposition have been identified around the cell surface: Cell lumens, cell wall, guard cells, intercellular spaces, root endodermal cells regions and bulliform cells [7,9,22,25]. Silicon is also found in the upper and lower epidermis of leaves as silica bodies that eventually constitute a cuticle-silica double layer [7,21,24,26,27].

The beneficial role of Si in citrus has been demonstrated in only a few studies. Si fertilisation has been reported to increase fruit yield, accelerate growth by 30–80% and fruit ripening by two to four weeks [28]. A similar study conducted in grapefruit revealed that calcium silicate slag fertilisation increased root and shoot mass by 19–40% [29]. Additionally, [30] reported an increase of 14–41% in tree height and 31–48% increase in shoot mass for Valencia trees.

In greenhouse studies, potassium silicate (K_2_SiO_3_) application improved fresh shoot mass by 30–40% in one-year-old and two-year-old sweet orange (*Citrus sinensis* (L.) Osbeck) trees over a six-month period [31]. The aim of this study was to examine the area of Si deposition in two Si accumulating citrus species.

## 2. Results

### 2.1. Si Accumulation in Citrus Species

**Figure 1:**
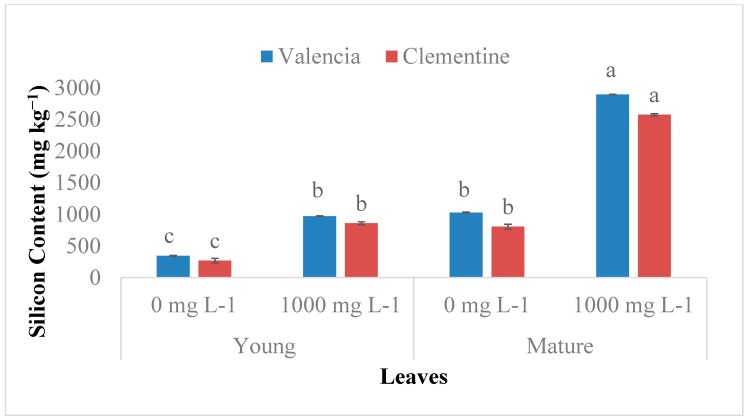
Si accumulation in young (new) and mature (old) leaves of two citrus species. Bars sharing a letter are not significantly different. Data are means ± standard errors.

**Figure 2:**
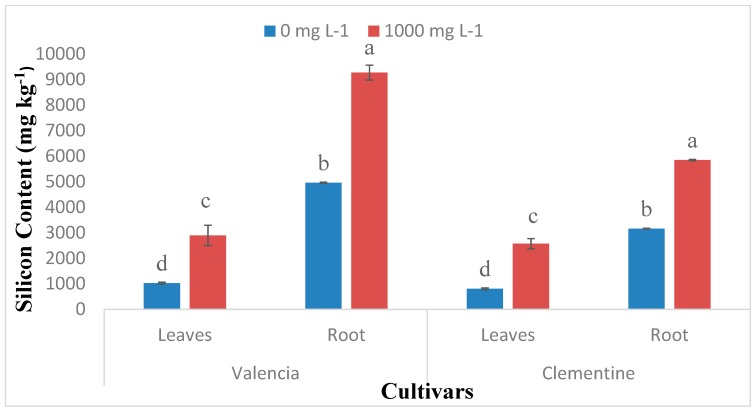
Si accumulation in mature leaves and roots of two citrus species. Bars sharing a letter are not significantly different. Data are means ± standard errors.

Si uptake increased significantly with leaves age in both Valencia and Clementine species regardless of Si treatment (Figure 1). The old leaves from plants drenched with 1000 mg L^−1^ had a significantly higher Si content compared to new leaves. For both species, drenching with 1000 mg L^−1^ resulted in significantly higher Si contents in both roots and leaves (Figure 2). Additionally, roots Si accumulation were significantly higher than leaves regardless of Si treatment in both citrus species.

### 2.2. Scanning Electron Microscopy with Energy Dispersive X-ray Analysis

**Figure 3:**
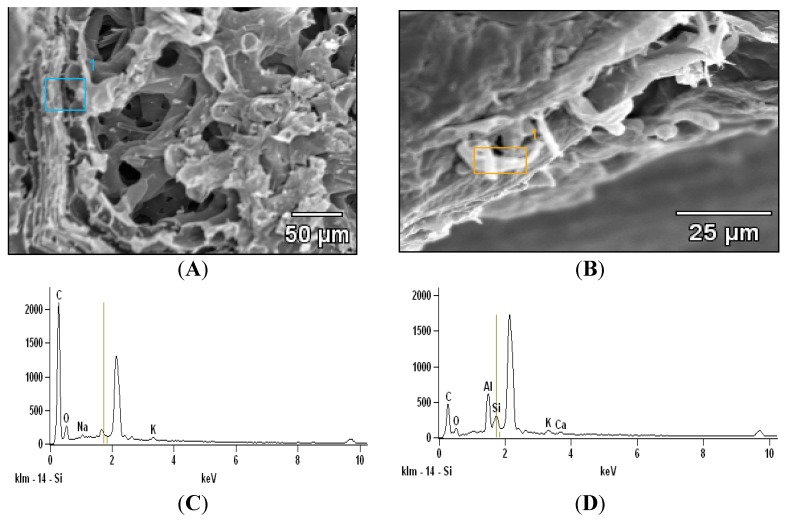
Scanning electron microscopy images of (**A**) −Si and (**B**) +Si mature leaves, white granules are areas of silicon detection. The selected areas (blue and orange blocks) were examined for elemental composition by energy dispersive X-ray analysis (EDAX) (**C**) −Si leaves (**D**) + Si leaves.

Scanning electron microscopy investigation in citrus leaves demonstrated the absence of silica bodies in non-treated Si leaves (Figure 3A). While silica bodies (granules) were present in epidermal cells of Si-treated leaves (Figure 3B). The elemental analysis using EDAX demonstrated Si presence in Si treated leaves while negligible amounts of Si were found in Si untreated leaves (Figure 3C,D).

### 2.3. Environmental Scanning Electron Microscopy (ESEM)

**Figure 4:**
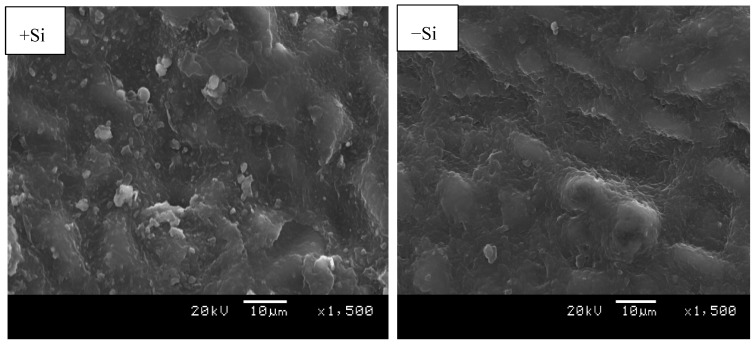
Environmental scanning electron microscopy (ESEM) micrographs of +Si and -Si matured citrus leaves, the white granules are areas of silicon detection on the adaxial leaf surfaces.

**Figure 5:**
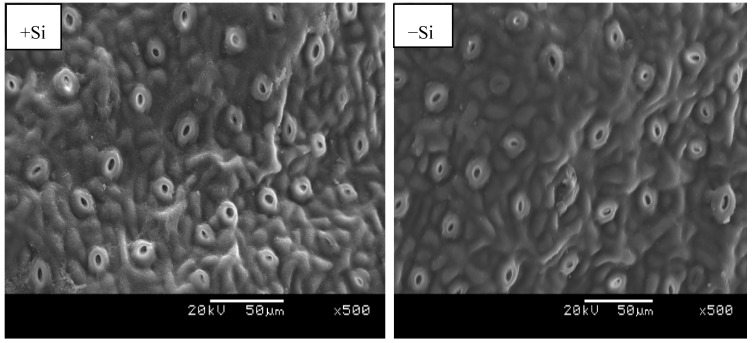
ESEM micrographs of matured citrus leaves subjected to +Si and −Si stomata abaxial leaf surfaces.

Environmental scanning electron microscopy (ESEM) was used to investigate Si presence in adaxial and abaxial leave surfaces. Si was located on the adaxial surface as white granules; more granules were found in Si treated leaves compared to the control (Figure 4). In the investigation of the abaxial region where stomata are found on citrus leaves, there was no Si deposit identified irrespective of the Si treatment (Figure 5). Despite the evidence of Si presence in the epidermal cell, the ESEM of the adaxial leaf surface did not provide the exact area of Si deposit within the cell structure.

### 2.4. Light Microscopy

**Figure 6:**
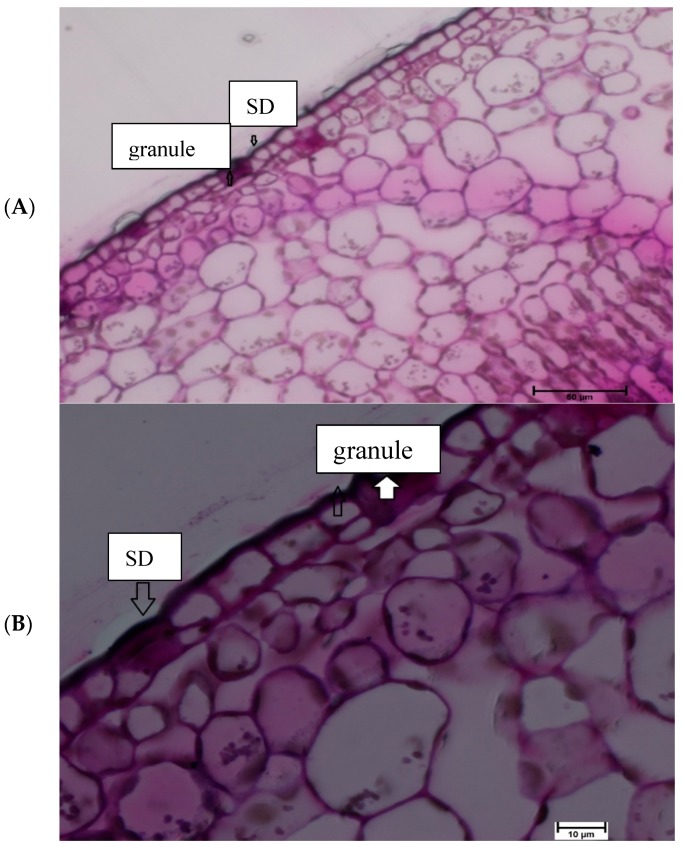
Si treated matured citrus leaves subjected to light microscopy lower epidermis surface at (**A**) 40×, and (**B**) 100× magnification: Arrows in the diagram represent silica granules in the epidermal surface and Si deposit in outer cell regions constitute the cuticle silica double layer (SDL).

**Figure 7:**
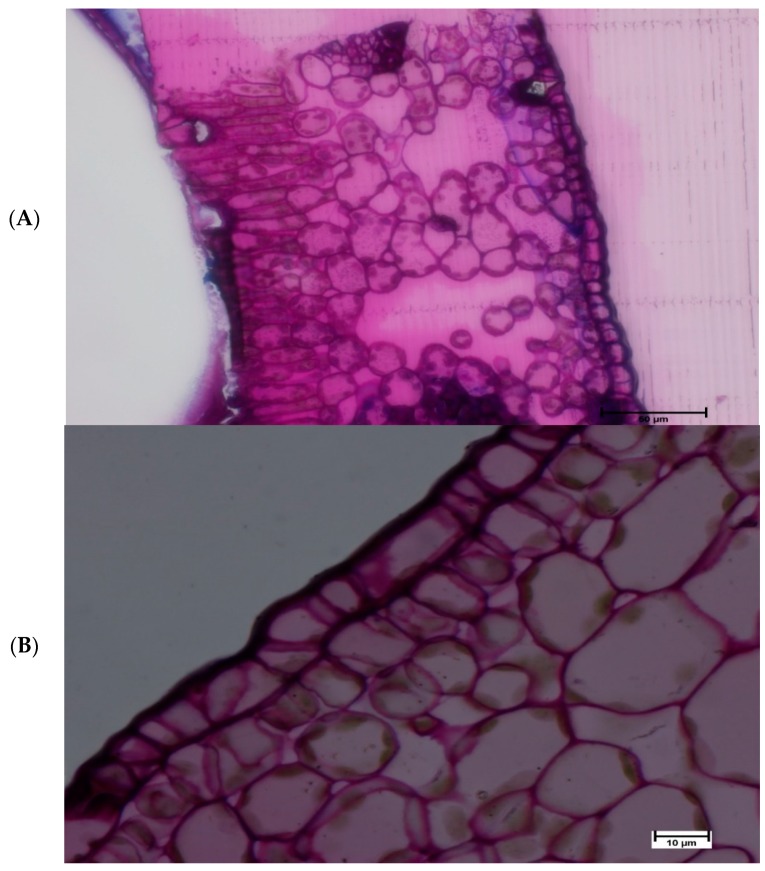
Si non-treated matured citrus leaves subjected to light microscopy (**A**) 40× and (**B**) 100× magnification: No silica deposit was located.

Light microscopy investigation provided further detail with regards to Si deposits in the cell structure. In the current study, cell structures were examined in both 40 and 100× magnification, the later provides better view. The results demonstrated that Silica granules were located on the lower epidermis cell of Si treated leaves. Additionally, silica deposits were found on the outer cell surface that constitutes a Si double layer in treated Si leaves (Figure 6). With respect to non-treated Si leaves no Si deposits were located on the leaf surface (Figure 7).

## 3. Discussion

The accumulation of Si in plants is related to several factors such as transpiration, species, leaf age, external Si supply, transporters and root uptake ability [4,6,11,13,14,17,32,33]. Si uptake significantly increased in both cultivars treated with Si. These results agree with previous studies conducted in tomato [34]; rice [6,7]; citrus [29,30,31]; forage grass [23] and cucumber [35] that demonstrated the Si accumulation rate to be linked to external Si concentration. In addition, the plant species role in Si accumulation is evident in higher plants [4,7,8]. Monocots are classified as active Si accumulator based on their silicon content in the range of 5–10 g kg^−1^ in dry weight [4,7,8]. While dicots are classified as a passive accumulator with Si content of less than 5 g kg^−1^ [8,36]. Our findings indicated that silicon content was less than 5 g kg^−1^ which is typical of Si passive accumulator [8,36].

In this study, the Si content significantly increased with age in the two citrus species irrespective of Si application level. This finding supports the statement that Si is not relocated within citrus plants as demonstrated by [7,31]. A similar trend in Si accumulation was found in other studies conducted on the leaves of bamboo (*Sasa veitchii*) and banana (*Musa acuminata*), Si concentration increased with leaf age, even after maturation [37,38]. Implying that the silica deposit is immobile within the plant and cannot be translocated to new leaves [7,8]. In addition, this supports the hypothesis of a passive uptake stated in forage grass *(Brachiaria brizantha*) plants due to Si accumulation not only during active growth stage but even after maturation [23,38].

The Si accumulation pattern in citrus leaves and roots were not affected by the Si supply dose, this corroborates with [39] who found that the Si accumulation pattern was not affected by the Si supply. In the current study, Si accumulation was significantly higher in the root than leaves. This result agrees with [29] who demonstrated a similar pattern of Si accumulation and speculated on the poor translocation of Si in citrus plants. In the study conducted on Si nutrition in tomato and bitter gourd the former demonstrated similar accumulation pattern whilst, in the latter shoot Si accumulation was higher than in the roots [32]. Another trend was observed by [40] whereby Si accumulation was higher in the shoot comparatively to the root in species with an active Si uptake. A similar trend was also observed in rice whereby 90% of Si taken up by the roots was translocated to the shoot [12]. Moreover, similar trends were observed in low-Si supplied banana [37] and rice [7] Si nutrition studies in Japan. This suggests the involvement of a passive uptake in citrus species.

The undetectable level of Si in citrus fruits further supports the hypothesis of non-translocation of Si within the plants due to the non-existence of a phloem mobility of Si in the citrus plants. This statement is sustained by the fact that no study has reported the occurrence of Si in the phloem, indicating Si cannot be relocated within the plant [37,41,42]. Phloem movement is bidirectional and nutrient translocation takes place within the plant which is not evident in the Si uptake [31,43]. However, Si transportation takes place via the xylem and is strongly dependent on transpiration [43].

Si deposition in plant cells depends on a number of factors, such as pH, Si concentration applied [44]. When the pH is lower than seven, which is the usual range inside cells, combined silica particles form chained oligomers due to weak electrostatic repulsion. However, at higher pH, the form condensed disordered polymers [45]. In the current study, Si deposition was only found in adaxial surfaces. Similar results were observed in shoot tissue of tropical forage grass (*Brachiaria brizantha*) which was classified as a passive uptake species [23]. While in other previous studies conducted on bamboo and sugarcane classified as active uptake Si accumulator, Si deposit were found on the abaxial surface [24,46]. Moreover, in rice a typical active Si accumulator more Si deposit were found in both leaf surfaces [44]. This implies that silica deposition in specific cell structure depends on leaf side (adaxial or abaxial surfaces) and plant species [23].

Our results demonstrated that silica deposition was only observed on the lower epidermis surface. Previous studies in rice and grass have determined more silica deposit in the upper epidermis compared to the lower epidermis [23,47]. Meanwhile, in sugarcane higher silica deposit was found in the lower epidermis [48]. This suggests that the silica deposit in the epidermis (lower or upper) depends on the number of silica cells present at the deposition site, which is specific to each plant species.

In the current study, silica polymerization as granules was identified in the epidermal cell of Si-treated citrus leaves. This corroborates with previous studies that have identified silica bodies in the epidermis surface of rice, grass and bamboo [7,21,24,26,27,44]. Some studies reported that silica deposit in the epidermal cell regions, which is the termini of transpiration stream provides substantiate evidence of transpiration involvement in Si deposition [2,7,27]. Silica deposit in outer cell surfaces that constitute cuticle Si double layer in Si-treated plants in this study has also been previously identified in Si nutrition studies conducted in *Brachiaria brizantha* and *Oryza Sativa* [7,9,23,44,49]. It is likely that Si incorporates cell surface as an organo-silicon compound made up with lignin and carbohydrate that provides a physical barrier against abiotic and biotic has previously suggested in rice [50,51]. Moreover, Si distribution along the epidermal cells in this study resembles the pathway of transpiration flow in the cell walls and intercellular space (apoplasm) transported into the plasma membrane and its trajectory to the xylem [7,43,52]. The presence of barriers in the apoplasmic movement such as endodermis cells and casparian bands implies the possible involvement of transporters Lsi1 which is known to facilitate Si passive uptake across the plasma membrane and plant cells [33,43].

## 4. Materials and Methods

### 4.1. Plant Material

Two citrus species Valencia ‘Delta’ and Clementine ‘Nules’ were used for the study. These are two widely cultivated commercial citrus cultivars in South Africa selected to compare two different citrus species namely: Orange and soft citrus (mandarins) [53].

### 4.2. Silicon Uptake Experiment

Two-year citrus seedlings were transferred in 10 L pots containing an artificial growing medium of Coir-Perlite 60–40% supplemented via root application with formulation of N, P and K at the concentration (85–115–40 kg ha^−1^) which represents fertiliser recommendation for two-year citrus trees [54]. N-P-K were applied in the form of ammonium nitrate (NH_4_NO_3_), potassium dihydrogen phosphate (KH_2_PO_4_) and potassium nitrate (KNO_3_). During this period, the water holding capacity was determined by watering the selected pot till drainage and weighed to determine subsequent irrigations. Each pot was irrigated to field capacity with 800 mL of distilled water every three days. The trees were left to acclimatize for three weeks prior to the Si uptake study.

The Si uptake experiment was performed for three months (January–April 2014 and 2018) in the glasshouse at the Experimental Farm of the University of Pretoria (S25° 44′ E28° 15′) on two-year-old citrus trees namely: ‘Delta’ Valencia oranges (*Citrus sinensis*) and ‘Nules’ Clementine (*Citrus reticulata*). The two species grafted on Carrizo citrange rootstocks were grown in 10 L pots and drenched once a month over three months with commercially available potassium silicate (1000 mg L^−1^) and in the control (0 mg L^−1^) the K introduced by K_2_SiO_3_ was corrected by adding K_2_O to the trees. Each treatment consisted of six replications (in groups of three plants per replicate), the 72 trees were organised in a randomised complete block design. These pots were kept in a climatically controlled greenhouse on a rotary table to minimize the effect of climatic differences on the plants. A month after the third drenching application, plant leaves collected were separated into young (new), and mature (old) leaves and roots collected 10 mm from the root base [55]. These samples were washed in distilled water to remove all impurities and grind for the Si analysis determined using ICP-OES.

### 4.3. Si Analysis

Polypropylene and Teflon containers were used for the preparation and digestion of plant samples to minimize contamination risks when using glassware. All containers were rinsed with NaOH (10%) prior to use. Leaf and root samples (500 mg) were placed in Teflon microwave digestion tubes and 3 mL of 65% HNO_3_ (AR grade) was added. The Teflon tubes were capped and left to stand for 5 min to thoroughly wet the sample. Then 2 mL of 30% H_2_O_2_ (AR grade) were added to the tubes and left overnight. The samples were placed in the microwave unit to the ramping temperature of 180 °C for 30 min and the acid digestion step conducted. NaOH (10%) solution was then added to the tubes and they were returned to the microwave system for the second heating step to solubilise amorphous Si. The contents of the Teflon tubes were transferred into a plastic beaker to minimize Si contamination then neutralized with HNO_3_ (2 M), using phenolphthalein as an indicator, and then diluted to 250 mL in a volumetric flask. A 10 mL sample was taken for Si determination with Inductively Coupled Plasma Optical Emission Spectrometry (ICP-OES, Varian Liberty 200); fitted with a hydrofluoric acid (HF) resistant torch, Sturrman Master spray-chamber and V Groove nebulizer assembly. The plasma power was 1000 W with a plasma flow rate of 15 L min^−1^ and an integration time of 1 s. Two Si sensitive wavelengths, 251.611 nm and 288.158 nm were used to detect Si. The wavelength 251.611 nm was selected as the most sensitive wavelength based on its higher correlation with the calibration curve. Matrix interferences were accounted for by preparing the sample and standard in a similar matrix.

### 4.4. Data Collection

#### 4.4.1. Microscopic analysis

##### Scanning Electron Microscopy (SEM) & EDAX Analysis

Silicon deposits were investigated in leaf surface with an electron microscopy. Scanning electron microscopy (SEM) was used to determine and identify Si precipitates within the leaf. Mature citrus leaves were collected after three months of Si application; washed with distilled water, wiped and then fixed with glutaraldehyde (primary fixation) for overnight and osmium tetroxide (post fixation) for 1 h. This was followed by wash with 0.05 M sodium cacodylate buffer and then subjected to dehydration series in ethanol. The specimens were placed in Hexamethyldisilazane left to air dried overnight and mounted on a stub using a carbon double-sided tape and sputter coated with gold using EMTECH K550X Coater and viewed at 3 kV with Zeiss Crossbeam 540 FEG scanning electron microscope. The energy dispersive analysis was conducted with an SEM-EDAX (XL30, Phillips, Eindhoven, Holland).

##### Environmental Scanning Electron Microscopy (ESEM)

Environmental scanning electron microscopy (ESEM) (XL30, Phillips, Eindhoven, Holland) was used to determine the Si content in citrus leaves adaxial and abaxial epidermal tissues (Valencia and Clementine). Mature citrus leaves were collected a month after the last drench; washed with distilled water and wiped before one square centimetre of leaf was excised with a razor blade and coated with gold, mounted on a carbon planchette and viewed on an ESEM instrument. The operating settings were: Voltage energy of 20 keV, with pump detection of 500 µm, the diffusion pump was set to a pressure between 1 and 2 Torr, wet mode, purge custom, spot size 6, room temperature (approximately 20 °C), working distance of 50 µm and the mechanical pump had a pressure of 10 mm (Hg). A gaseous secondary detector was used to determine Si.

##### Light Electron Microscopy

Plant material (approximately 2 mm^2^) were cut from the region between the leaf margin and midrib of the middle sections. The tissues were fixed in 3% glutaraldehyde for 24 h at 1 °C. The tissues were then washed in 0.05 M Sodium Cacodylate buffer for 30 min. The samples were then post-fixed with 2% osmium tetroxide with pH 7.2 for overnight. After this, they were washed twice in a 0.05 M Na-cacodylate buffer for 30 min.

The specimens were dehydrated through a series of ethanol 10, 30 and 50% (v/v) ethanol, for 15 min at each dehydration step. The samples were left overnight at 50% (v/v). The next day, the samples were dehydrated through 70, 90, 100% (v/v) ethanol at 15 min intervals between each step. The dehydration series were completed with two rinses of 15 min each in absolute alcohol. The low viscosity (LV) resin is not miscible with alcohol and, therefore, from the 100% alcohol we proceeded to the solvent propylene oxide in two rinses, the tissues were left overnight in propylene oxide. In order to ensure adequate infiltration of the material with the embedding mixture the tissues were left over night in 25:375 LV Resin/propylene oxide. The next day, the tissues were placed in 50:50 LV resin /propylene oxide for overnight, 75:25 LV resin/ propylene oxide for 2 h and twice into 100% LV Resin for 1 h.

The tissues were finally placed in approximately labelled moulds and polymerized for 16 h at 60 °C. After removal of the tissues from the oven the tissues could cool at room temperature before sectioning. Selected tissue regions were sectioned in the range of 0.5–1.0 μm. Thick sections were done using the LKB ULTRAMICROTOME III (Stockholm, Sweden) with a knife clearance angle set at 5° to produce purple sections.

The sections were picked up and mounted onto a glass slide and stained with Ladd’s stain for 20 s and rinsed in distilled water. The tissues were viewed using the Olympus BH2 light microscope at 40× and 100× magnification.

#### 4.4.2. Statistical Analysis

The collected data was subjected to the analysis of variance by using the Statistical Analysis System software (SAS) version 9.4 (Cary, NC, USA) to determine treatment mean effects. Differences between treatments were determined using Fisher’s Least Significant Difference (LSD) at 5% level of significance.

## 5. Conclusions

Si is deposited in lower epidermis cell wall regions as granules and silica deposit on the outer cell surface that constitute the cuticle-silica double layer in both species. Therefore, water loss through the epidermis could be reduced especially when plants are exposed to unfavourable abiotic conditions. The absence of Si in the stomata implies the non-involvement of Si in the stomata structure. Our study supports the hypothesis that the Si deposition in citrus takes place passively through the transpiration stream implying that Si provides alleviation in stress conditions only when continuously supplied to the plants. There is also a possible involvement of transporters in the passive uptake process which requires further investigation.

## References

[1] Birchall J. (1995). The essentiality of silicon in biology. Chem. Soc. Rev..

[2] Ma J.F., Yamaji N. (2006). Silicon uptake and accumulation in higher plants. Trends Plant Sci..

[3] Jones L., Handreck K. (1967). Silica in soils, plants, and animals. Advances in Agronomy.

[4] Epstein E. (1999). Silicon. Ann Rev Plant Biol..

[5] Haynes R.J. (2014). A contemporary overview of silicon availability in agricultural soils. J. Plant Nutr. Soil Sci..

[6] Mitani N., Ma J.F. (2005). Uptake system of silicon in different plant species. J. Exp. Bot..

[7] Ma J.F., Takahashi E. (2002). Soil, Fertilizer, and Plant Silicon Research in Japan.

[8] Tubana B.S., Babu T., Datnoff L.E. (2016). A review of silicon in soils and plants and its role in US agriculture: History and future perspectives. Soil Sci..

[9] Raven J.A. (2003). Cycling silicon–The role of accumulation in plants. New Phytol..

[10] Liang Y., Si J., Römheld V. (2005). Silicon uptake and transport is an active process in Cucumis sativus. New Phytol..

[11] Yamaji N., Mitatni N., Ma J.F. (2008). A transporter regulating silicon distribution in rice shoots. Plant Cell.

[12] Ma F.J., Yamaji N., Mitani-Ueno N. (2011). Transport of silicon from roots to panicles in plants. Proc. Jpn. Acad. Ser. B.

[13] Mitani N., Yamaji N., Ago Y., Iwasaki K., Ma J.F. (2011). Isolation and functional characterization of an influx silicon transporter in two pumpkin cultivars contrasting in silicon accumulation. Plant J..

[14] Takahashi E., Ma J., Miyake Y. (1990). The possibility of silicon as an essential element for higher plants. Comments Agric. Food Chem..

[15] Yamaji N., Chiba Y., Mitani-Ueno N., Ma J.F. (2012). Functional characterization of a silicon transporter gene implicated in silicon distribution in barley. Plant Physiol..

[16] Ma J., Yamaji N. (2008). Functions and transport of silicon in plants. Cell. Mol. Life Sci..

[17] Rao G.B., Susmitha P. (2017). Silicon uptake, transportation and accumulation in Rice. J. Pharmacogn. Phytochem..

[18] Chiba Y., Mitani N., Yamaji N., Ma J.F. (2009). HvLsi1 is a silicon influx transporter in barley. Plant J..

[19] Montpetit J., Vivancos J., Mitani-Ueno N., Yamaji N., Rémus-Borel W., Belzile F., Ma J.F., Bélanger R.R. (2012). Cloning, functional characterization and heterologous expression of TaLsi1, a wheat silicon transporter gene. Plant Mol. Biol..

[20] Mitani N., Chiba Y., Yamaji N., Ma J.F. (2009). Identification and characterization of maize and barley Lsi2-like silicon efflux transporters reveals a distinct silicon uptake system from that in rice. Plant Cell.

[21] Motomura H., Fujii T., Suzuki M. (2000). Distribution of silicified cells in the leaf blades of Pleioblastus chino (Franchet et Savatier) Makino (Bambusoideae). Ann. Bot..

[22] Motomura H., Fujii T., Suzuki M. (2004). Silica deposition in relation to ageing of leaf tissues in Sasa veitchii (Carriere) Rehder (Poaceae: Bambusoideae). Ann. Bot..

[23] De Melo S.P., Monteiro F.A., De Bona F.D. (2010). Silicon distribution and accumulation in shoot tissue of the tropical forage grass Brachiaria brizantha. Plant Soil..

[24] Motomura H., Fujii T., Suzuki M. (2006). Silica deposition in abaxial epidermis before the opening of leaf blades of *Pleioblastus chino* (Poaceae, Bambusoideae). Ann. Bot..

[25] Heckman J. (2013). Silicon: A beneficial substance. Better Crops.

[26] Richmond K.E., Sussman M. (2003). Got silicon? The non-essential beneficial plant nutrient. Curr. Opin. Plant Biol..

[27] Kaufman P.B., Dayanandan P., Franklin C., Takeoka Y. (1985). Structure and function of silica bodies in the epidermal system of grass shoots. Ann. Bot..

[28] Taranovskaia V.G. (1939). The silicification of subtropics greenhouse and plantations. Sov. Subtrop..

[29] Matichenkov V., Calvert D., Snyder G. (1999). Silicon fertilizers for citrus in Florida. Proc. Fla. State Hortic. Soc..

[30] Matichenkov V., Bocharnikova E., Calvert D. (2001). Response of citrus to silicon soil amendments. Proc. Fla. State Hortic. Soc..

[31] Wutscher H. (1989). Growth and mineral nutrition of young orange trees grown with high levels of silicon. HortScience..

[32] Heine G. (2005). Silicon Nutrition and Resistance against Pythium Aphanidermatum of *Lycopersicon Esculentum* and *Mormodica Charantia*. Ph.D. Thesis.

[33] Ma J.F., Yamaji N. (2015). A cooperative system of silicon transport in plants. Trends Plant Sci..

[34] Marodin J.C., Resende J.T., Morales R.G., Silva M.L., Galvão A.G., Zanin D.S. (2014). Yield of tomato fruits in relation to silicon sources and rates. Hortic. Bras..

[35] Adatia M., Besford R. (1986). The effects of silicon on cucumber plants grown in recirculating nutrient solution. Ann. Bot..

[36] Liang Y., Hua H., Zhu Y.G., Zhang J., Cheng C., Römheld V. (2006). Importance of plant species and external silicon concentration to active silicon uptake and transport. New Phytol..

[37] Henriet C., Draye X., Oppitz I., Swennen R., Delvaux B. (2006). Effects, distribution and uptake of silicon in banana (Musa spp.) under controlled conditions. Plant Soil.

[38] Motomura H., Mita N., Suzuki M. (2002). Silica accumulation in long-lived leaves of Sasa veitchii (Carrière) Rehder (Poaceae–Bambusoideae). Ann. Bot..

[39] Jones L., Handreck K. (1969). Uptake of silica by Trifolium incarnatum in relation to the concentration in the external solution and to transpiration. Plant Soil..

[40] Ma J.F., Mitani N., Nagao S., Konishi S., Tamai K., Iwashita T., Yano M. (2004). Characterization of the silicon uptake system and molecular mapping of the silicon transporter gene in rice. Plant Physiol..

[41] Heine G., Tikum G., Horst W.J. (2005). Silicon nutrition of tomato and bitter gourd with special emphasis on silicon distribution in root fractions. J. Plant Nutr. Soil Sci..

[42] Casey W., Kinrade S., Knight C., Rains D., Epstein E. (2004). Aqueous silicate complexes in wheat, *Triticum aestivum* L.. Plant Cell Environ..

[43] Marschner H. (2012). Marschner’s Mineral Nutrition of Higher Plants.

[44] Zhang C., Wang L., Zhang W., Zhang F. (2013). Do lignification and silicification of the cell wall precede silicon deposition in the silica cell of the rice *(Oryza sativa* L.) leaf epidermis?. Plant Soil..

[45] Coradin T., Lopez P.J. (2003). Biogenic silica patterning: Simple chemistry or subtle biology?. ChemBioChem.

[46] Sakai W.S., Sanford W. (1984). A developmental study of silicification in the abaxial epidermal cells of sugarcane leaf blades using scanning electron microscopy and energy dispersive X-ray analysis. Am. J. Bot..

[47] Takahashi N., Kato Y., Isogai A., Kurata K. (2006). Silica distribution on the husk epidermis at different parts of the panicle in rice (*Oryza sativa* L.) determined by X-ray microanalysis. Plant Prod. Sci..

[48] Naidoo P., McFarlane S., Keeping M., Caldwell P. Deposition of silicon in leaves of sugarcane (Saccharum spp. hybrids) and its effect on the severity of brown rust caused by Puccinia melanocephala. Proceedings of the Annual Congress-South African Sugar Technologists’ Association.

[49] Schurt D.A., Cruz M.F., Nascimento K.J., Filippi M.C., Rodrigues F.A. (2014). Silicon potentiates the activities of defense enzymes in the leaf sheaths of rice plants infected by Rhizoctonia solani. Trop. Plant Pathol..

[50] Inanaga S., Okasaka A., Tanaka S. (1995). Does silicon exist in association with organic compounds in rice plant?. Soil Sci. Plant Nutr..

[51] Kim S.G., Kim K.W., Park E.W., Choi D. (2002). Silicon-induced cell wall fortification of rice leaves: A possible cellular mechanism of enhanced host resistance to blast. Phytopathol..

[52] Hodson M., Sangster A. (1989). Silica deposition in the inflorescence bracts of wheat (Triticum aestivum). II. X-ray microanalysis and backscattered electron imaging. Can. J. Bot..

[53] Saunt J. (2000). Citrus Varieties of the World.

[54] FSSA-MVSA (2007). Fertiliser Handbook of South Africa.

[55] Lux A.L.M., Abe J., Tanimoto E., Hattori T., Inanaga S. (2003). The dynamics of silicon deposition in the sorghum root endodermis. New Phytol..

